# Endocrine Disrupting Chemicals: Current Understanding, New Testing Strategies and Future Research Needs

**DOI:** 10.3390/ijms22020933

**Published:** 2021-01-19

**Authors:** Maria E. Street, Karine Audouze, Juliette Legler, Hideko Sone, Paola Palanza

**Affiliations:** 1 Division of Paediatric Endocrinology and Diabetology, Paediatrics, Department of Mother and Child-AUSL of Reggio Emilia-IRCCS, 42123 Reggio Emilia, Italy; 2 INSERM UMR S1124, Université de Paris, 75006 Paris, France; karine.audouze@u-paris.fr; 3 Institute for Risk Assessment Sciences (IRAS), Utrecht University, 3508 TD Utrecht, The Netherlands; j.legler@uu.nl; 4 Environmental Health and Prevention Research Unit, Yokohama University of Pharmacy, Yokohama 245-0066, Japan; hideko.sone@yok.hamayaku.ac.jp; 5 Unit of Neuroscience, Department of Medicine and Surgery, University of Parma, 43125 Parma, Italy; paola.palanza@unipr.it

Endocrine disrupting chemicals (EDCs) are exogenous chemicals which can disrupt any action of the endocrine system, and are an important class of substances which play a role in the Developmental Origins of Health and Disease (DOHaD) [1]. Until now most of the data on the harmful effects of EDCs come from animal studies [2] focusing on a limited number of chemicals and endpoints, and prospective human studies have been carried out but are inevitably limited both in number and scope. Effects of EDCs can occur at any age but when these take place at an early life stage, they may alter gene expression, protein content, cell number, cell size, organ size and function besides other effects, thereby playing a role in susceptibility to disease and dysfunctions later in life because of altered “programming” in utero.

There is an urgent need for further studies on EDCs, both in animal and human epidemiological studies, but also via innovative computational approaches. In addition, data on human exposure during pregnancy, at birth and postnatally are scarce, and the effect of EDCs on the timing and progression of puberty, and on fertility are still preliminary. Furthermore, the first studies focused attention on single compounds whereas it is increasingly necessary to focus on exposure to mixtures of EDCs which reflect better real life.

While the original studies of EDCs focused mainly on chemicals that could interfere with (male) reproduction mainly through estrogen and androgen pathways, it is clear that the multiple endocrine pathways can be affected by EDCs, both in males and females. The Parma Consensus in 2015 highlighted how EDCs may disrupt metabolic systems during critical windows of development with an impact on non-communicable diseases such as obesity, diabetes and the metabolic syndrome [3]. Similarly, robust evidence from experimental studies in animal models, together with fewer human epidemiological studies, has indicated that brain and behaviour are sensitive targets of endocrine disruption [2]. Across mammalian species, developmental exposure to EDCs has been shown to affect emotional, motor, cognitive, social, sexual and parental behaviour [2,4]. Findings in human studies are highly heterogeneous, but reveal an overall negative impact of gestational exposure to EDCs on child neurobehavioural development [2,4]. In both animal and human studies, current evidence points to sex-specific effects of developmental exposure to EDCs depending upon type of chemicals and the specific endpoints examined [4]. Nonetheless, a major outcome emerging from the recent literature is that animal and human studies have a reasonable degree of consistency, indicating that epidemiological studies on child development substantially reflect findings in animal models [4]. This is a critical issue to highlight the value of experimental animal models to predict human findings and for assessing hazards to humans posed by EDCs.

This Special Issue, entitled ‘Advances in the Research of Endocrine Disrupting Chemicals 2.0’ comprises state-of -the art reviews, original articles, and project reports that introduce new research in the field. The manuscripts in this Special Issue address three main themes: (1) Advancing our understanding of the effects of EDCs, using experimental studies in animal and cell models; (2) Reviews of the literature on the less known effects of EDCs, and (3) Introduction to new projects that have the goal of developing better testing strategies for the lesser-studied EDCs, and for identifying potential non-characterized EDC compounds.

## 1. Experimental Studies

In this Special Issue, new understanding is shed on the potential health effect of different EDCs using various experimental models. Some studies indicate that Bisphenol A (BPA) analogues such as Bisphenol S (BPS) which are currently unregulated, and used in the plastic industry, are not safe alternatives for BPA. In ovines, BPS exposure impairs oocyte development competence with blastocyte rate reduction being observed [5]. In human granulosa cells, the administration of BPS leads to reduced secretion of oestradiol and progesterone which are observed after 48 h of exposure suggesting that the effects of chronic exposure might be similar [6]. As BPA crosses the placental barrier, and is known to have an effect on the expression of genes involved with fetal brain development, gene expression in the anterior hypothalamus was studied after exposure in mice highlighting the involvement particularly of genes that are currently thought to be key in Autism Spectrum Disorders [7]. An encouraging and new finding was that Resveratrol was able to prevent programmed hypertension induced by BPA exposure combined with high fat diet, opening the way towards possible future clinical applications using specific supplements, and moreover opening to the possibility of implementing reprogramming strategies for the future [8]. In male mice, BPA and Diethylstilbestrol exposure in the perinatal period and subsequent exposure in adult life to testosterone and oestradiol was related with dorsal prostate hyperplasia and prostatitis, providing proof for the “multiple hits” hypothesis to environmental exposure for the development of obstructive voiding disorders and prostate diseases [9]. Studies with BPA emphasize that sex is a fundamental variable in accounting for EDC effects on neural and behavioural development [4]. Maternal exposure of mice to a low dose of the plastic derivative bisphenol A (BPA) during the last week of pregnancy had long term, sex-dependent disruptive effects on brain noradrenergic system and on emotional behavioural responses to a novel environment, but did not alter sexual behavior [10]. BPA exposure altered affinity and density of alpha2-adrenergic receptors in the adult brain and it eliminated the sexual dimorphism in the density of the receptors in the medial preoptic area, thus suggesting that BPA may interfere with the sex-related organization of these receptors’ pharmacodynamics and expression [10].

In addition to studies with bisphenol analogues, new studies show that other classes of compounds may disrupt the endocrine system, such have metals and flame retardants. Lead (Pb) is an environmental toxicant that can interfere with hormone signalling and primarily affect the central nervous system (CNS). Developmental exposure of rats to a low concentration of Pb induced in the offspring behavioral and neural changes that were more pronounced in females than in males. While perinatal exposure to Pb induced alterations in neonatal motor activity and adult spatial memory retention in both sexes, only Pb-exposed females showed increased anxiety-like behavior, impairment in recognition memory, spatial memory accuracy and selective alterations in glutamatergic receptors distribution [10]. Exposure of Wistar rat dams to flame retardant mixtures indicates a negative effect during gestation and lactation on bone composition in male offspring only. At six months of age, effects were observed on mesenchymal stem cells, highlighting the effect of sex and how duration and time of exposure are important. It should also be considered that mixtures can also be formed when that single components are metabolized to several different metabolites [11].

## 2. State of the Art Reviews

This Special Issue includes a number of timely review articles. The detection methods and the mode of action of oestrogenic endocrine disruptors in placenta are extensively reviewed in order to provide guidance for human health keeping in mind that air, dust, cosmetics, food and water are sources of EDCs that are found in all body fluids (urine, serum, breast milk, amniotic fluid). Through damage to the placenta EDCs can lead to abortion, preeclampsia, altered fetal growth, congenital malformations and increased cancer risk [12]. Regarding placental and fetal growth, as well as in utero programming of metabolism and endocrine function, current knowledge highlights the need for the implementation of preventive measures for exposure addressed in particular to pregnant mothers [13]. 

Human studies considering an effect on the pathogenesis of type 1 diabetes mellitus are controversial, and certainly this is one of the fields requiring further studies due to the increasing incidence of diabetes worldwide in line with the actions proposed as necessary by the 2015 Parma Consensus Statement [3,14]. Recent data also indicate that contamination of water may be an important source of exposure to EDCs. Among these, attention must be drawn to disinfection by-products which are produced by the reaction of disinfection agents and organic and inorganic matter. Reproductive effects are reported to differ among species, and as previously stated, mixtures of contaminants should be studied as they mimic better normal environment exposure [15]. It should be noted that within the field of EDCs, the lack of statistical significance does not mean absence of effect, and that further studies and new scientific approaches, as integrating the exposome with the different “omics”, are needed, and a new field to be further explored is relationships between EDC exposure and “gut microbiome”. Gut dysbiosis is nowadays recognized as a cause of disease and metabolic derangement including conditions such as obesity, diabetes, neurobehavioural disorders and immunological and gastrointestinal alterations. Recent studies have shown changes in the gut microbiome induced by the exposure to EDCs leading to pathophysiological changes [13,16].

EDCs may also influence the timing and progression of puberty, and current evidence suggests that exposure to EDCs during puberty may predispose women to breast cancer later in life [17]. Importantly, the new research project ‘PEACH’ has recently started which will provide much needed information on human exposure to pesticides and idiopathic premature thelarche in girls from areas of intensive agriculture practice [18].

## 3. New Projects within the EURION Cluster

This Special Issue is also proud to introduce the eight new projects in the field of EDC research, which are part of the ‘European Cluster to Improve Identification of Endocrine Disruptors (EURION)’ (https://eurion-cluster.eu/). EURION is the result of projects granted from the European Commission’s Horizon 2020 Research and Innovation Programme Call SC1-BHC-27-2018—New testing and screening methods to identify endocrine disrupting chemicals (EDCs). EURION is funded €50 million by the European Commission, the largest public funding of this type of research in Europe. Each project in the cluster focusses on a different aspect of test method development in order to identify lesser-studied ED outcomes, i.e., outside of estrogenic and androgenic effects. The projects use an ‘adverse outcome pathway’ (AOP) framework, to develop tests that span molecular initiating events (including in silico predictive models and high throughput screening), key events in in vitro models, and measure adverse outcomes in rodents, zebrafish and humans. Three projects within the EURION focus on improved testing strategies for thyroid hormone disrupting chemicals: ERGO [19], ATHENA [20], and SCREENED [21], while three other projects focus on integrated approaches for the testing and assessment of metabolism disrupting chemicals: OBERON [22], EDCMET [23] and GOLIATH [24]. In addition, the FREIA project develops new tests for effects of EDCs on female reproduction [25] and the ENDpoiNTs project focusses on developmental neurotoxicity [26] and methods for elucidating EDC effects.

All works (state-of -the art reviews, original articles, and project reports) within this special issue ‘Advances in the Research of Endocrine Disrupting Chemicals 2.0’ are very complementary to each other, and introduce a good state-of-art of the current research in the area of EDCs. This highlights also information regarding some current gaps, such as the need of new testing strategies to assess suspected EDCs and to identify novel EDCs, as well as the development of studies to provide knowledge regarding their mode of action or adverse effects. Integrated together, all these interdisciplinary studies and researches (experimental methods, high throughput omics technologies, epidemiology and human biomonitoring studies, and advanced computational models), could provide useful insights to regulatory efforts to better characterize suspected EDCs, and their connection to health outcomes. The improvement of EDC related information is crucial for future regulatory measures to ensure a good protection of the EU and non-EU citizens and the environment.
